# Robot-assisted laparoscopic ipsilateral ureteroureterostomy for duplex kidneys in children: preliminary single-center experience

**DOI:** 10.3389/fped.2024.1470948

**Published:** 2025-01-06

**Authors:** Chao Yang, Chi Zhang, Yongsheng Cao, Qi-fei Deng, Changkun Mao

**Affiliations:** ^1^ Department of Urology, Anhui Provincial Children’s Hospital, Hefei, China; ^2^ Department of Oncology, Anhui Chest Hospital, Hefei, Anhui, China

**Keywords:** robotic surgery, duplex kidney, duplicated ureter, ipsilateral ureteroureterostomy, RAL-IUU

## Abstract

**Objective:**

This study evaluates the efficacy and safety of robot-assisted laparoscopic ipsilateral ureteroureterostomy (RAL-IUU) in treating children with duplex kidney ureteral malformations by detailing our early single-center experience.

**Materials and methods:**

We conducted a retrospective analysis of clinical data from 14 children with complete duplex kidney ureteral malformations treated with RAL-IUU at our institution from December 2021 to January 2024. Clinical data included patient demographics, surgical details, and postoperative outcomes.

**Results:**

The operation time averaged 128.71 ± 22.35 min in 14 cases, intraoperative blood loss was 7.57 ± 2.77 ml, drainage tube placement lasted for 3.14 ± 0.66 days, and hospital stay averaged 4.79 ± 0.70 days. Stent placement lasted 43.58 ± 6.33 days. Notable changes were observed in the upper moiety anterior-posterior diameter (APD) before and after surgery (23.84 ± 8.05 mm vs. 6.71 ± 2.20 mm, *P* < 0.001), diameter at the widest part of the upper moiety ureter (15.58 ± 6.07 mm vs. 4.61 ± 0.78 mm, *P* < 0.001), and split renal function of the upper moiety (12.28 ± 3.04% vs. 16.50 ± 2.75%, *P* < 0.001). Postoperative follow-up ranged from 6 to 18 months; during the period with a D-J stent, one case developed a urinary tract infection, one case had recurrent gross hematuria, and another child exhibited significant urinary irritative symptoms (frequency), with an abdominal plain film revealing that the D-J tube had descended completely into the bladder, and symptoms disappeared after the removal of the D-J tube. During the follow-up period post-D-J tube removal, none of the 14 children experienced a urinary tract infection again during the follow up period, urinary incontinence ceased.

**Conclusion:**

RAL-IUU provides an excellent surgical field and operating space, precise suturing, and minimal surgical trauma. Postoperatively, there is a reduction in renal pelvis and ureteral hydronephrosis, recovery of split renal function, and minimal complications all with rapid recovery. RAL-IUU is a safe and feasible treatment option for children with complete duplex kidneys.

## 1 Introduction

Duplex kidney represents a frequent congenital anomaly of the pediatric urinary system, exhibiting an incidence rate of 0.8%-1% and occurring more commonly in females than in males (1). The clinical presentations often include urinary tract infections, urinary incontinence, and abdominal pain (2). Many cases are identified during prenatal screenings and postnatal physical examinations. The presence of duplex kidney combined with ectopic ureteral orifices or vesicoureteral reflux (VUR) presents significant challenges for pediatric urologists, necessitating tailored treatment strategies based on the specific circumstances. Current therapeutic approaches primarily focus on preserving and salvaging the duplex kidney. Surgical interventions aimed at kidney preservation include ectopic ureter reimplantation, common sheath ureter reimplantation, and ipsilateral ureteroureterostomy (IUU) (3). The evolution of IUU surgery from open to laparoscopic and robot-assisted laparoscopic techniques has established it as both a safe and effective method (4–6).

However, literature on robot-assisted laparoscopic ipsilateral ureteroureterostomy (RAL-IUU) in pediatric cases remains scarce; this paper contributes by reporting on 14 instances of RAL-IUU at our center, assessing its safety and efficacy.

## 2 Materials and methods

### 2.1 Study design

This retrospective study was approved by our institution's Ethics Committee (Approval No.: EYLL-2024-008) and conducted in accordance with the Declaration of Helsinki (2013 revision). Written informed consent was obtained from the legal guardians of all eligible patients. We performed a retrospective analysis of clinical data from pediatric patients with complete bilateral ureteral duplication who underwent RAL-IUU treatment at our institution between December 2021 and January 2024. The collected data included patient demographics, surgical parameters, and postoperative outcomes. Statistical analyses were conducted using SPSS software version 23.0.The final results were analyzed and discussed.

### 2.2 Inclusion and exclusion criteria of study subjects

Inclusion Criteria: Presence of a complete duplex kidney; renal duplex with functioning upper moiety associated with ectopic ureter causing obstruction or urinary incontinence or VUR while the lower moiety is normal without any pathology like VUR; symptoms including urinary incontinence, recurrent urinary tract infections, abdominal pain, progressive worsening of hydronephrosis in the upper moiety and ureter; dynamic renal scintigraphy (DRS) and Magnetic Resonance Urography (MRU) confirming the upper moiety viability for preservation. Exclusion Criteria: Incomplete duplex kidney; DRS indicating non-functionality of the upper moiety; the upper moiety with associated obstructive ureterocele, regardless of whether ureterocele incision has been performed; co-occurrence with other upper urinary tract malformations necessitating surgical intervention; VUR or obstruction in the ureter of the lower moiety.

### 2.3 Surgical techniques

The surgery was conducted by the same surgeon, who possesses extensive expertise in robot-assisted laparoscopic surgery.

Following successful general anesthesia, the patient was positioned in the lithotomy pose. A pediatric cystoscope was inserted through the urethra and advanced to the lower moiety ureteral opening on the affected side to place a D-J stent, after which the cystoscope was removed, leaving the catheter *in situ*. Subsequently, the patient was repositioned supine, with the affected side elevated by 30°. The surgical area was disinfected again and draped, a 10 mm vertical incision was made at the umbilicus, through which the umbilical ring was separated, and the peritoneum was opened to insert an 8 mm Trocar, establishing a pneumoperitoneum. Additional 8 mm Trocars were placed below the xiphoid and slightly contralateral to the umbilicus for the attachment and operation of the Da Vinci Si robotic arms. Robotic forceps were introduced, and a 5 mm Trocar was inserted in the contralateral lower abdomen as an auxiliary port.

**Figure 1:**
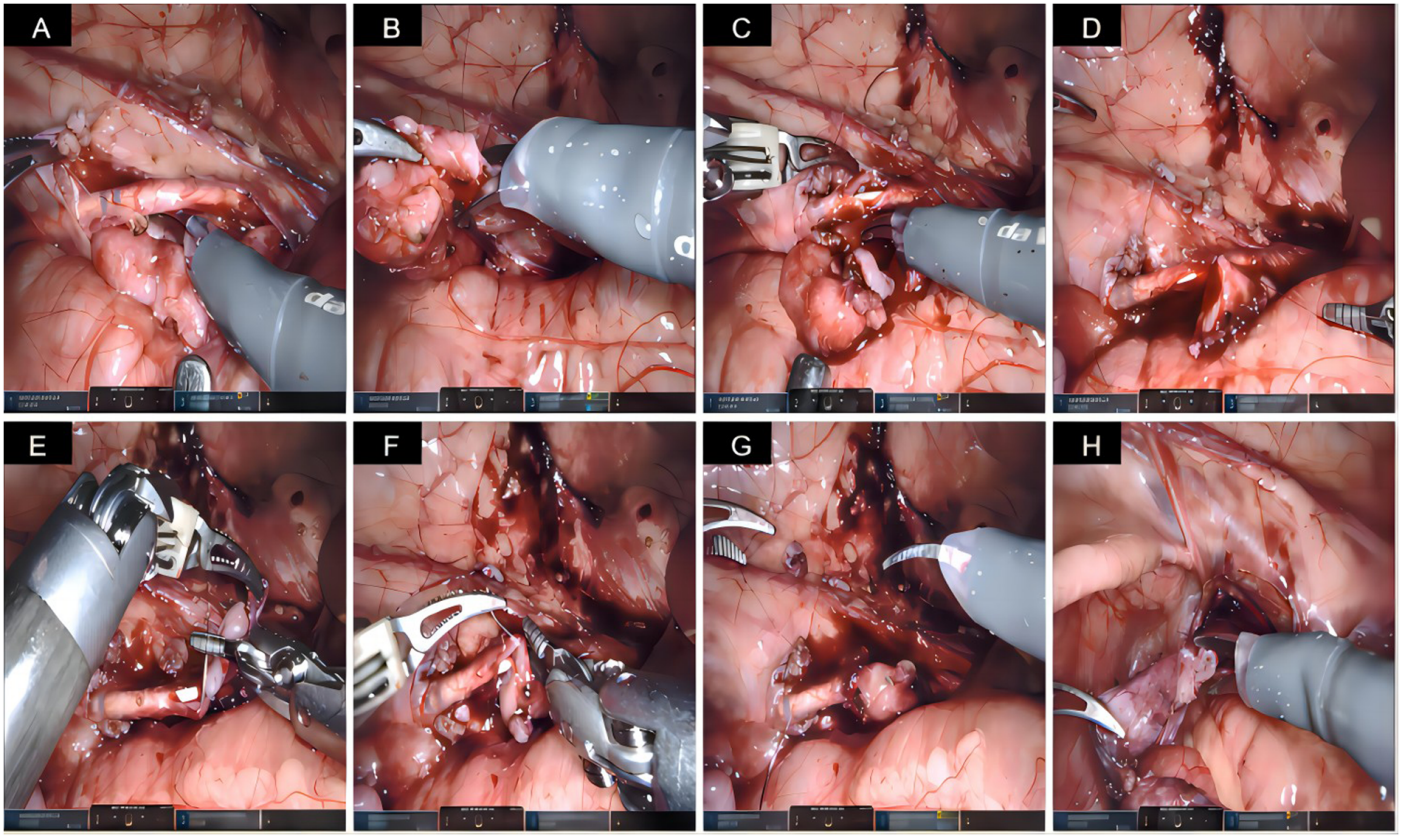
Surgical Steps. **(A)** Exposure of both ureters (the upper kidney ureter is dilated and tortuous, while the lower kidney ureter is not dilated but contains a D-J stent). **(B)** Transection of the upper kidney ureter anterior to the iliac vessels. **(C)** Longitudinal incision made on the side of the lower kidney ureter. **(D, E, F)** End-to-side anastomosis of the distal end of the upper ureter to the lower ureter. **(G)** Inspection of the anastomosis between the duplicated upper and lower ureters to ensure there is no tension, twisting, or urine leakage. **(H)** After mobilizing the upper ureter, the dilated segment is excised and the end is ligated.

The colon was displaced within the abdominal cavity, the paracolic gutter was opened using electrocautery scissors, and the ureter was dissected from the iliac vessels level to the mid-ureter, revealing two parallel ureters. The upper moiety ureter appeared dilated and tortuous, whereas the lower moiety ureter was not dilated and contained a visible D-J stent. Both duplicated ureters were fully dissected and separated. The upper moiety ureter was transected anterior to the iliac vessels, and if the ureter width exceeded 15 mm, it was trimmed and sutured to reduce the cross-sectional diameter to approximately 10 mm. A longitudinal incision equal in length to the width of the upper moiety ureter was made on the side of the lower moiety ureter. The distal end of the duplicated upper moiety ureter was anastomosed end-to-side with the lower moiety ureter. The anastomosis between the duplicated upper and lower moiety ureters was inspected to ensure there was no tension, twisting, or urine leakage. The upper moiety ureter was further dissected distally from the transection point, and the dilated section of the ureter below the iliac vessels was reduced by 1 cm, and the end was sealed with a double 4-0 absorbable suture. A drainage tube was positioned in the pelvis, the robotic system was withdrawn, and all incisions were sutured. The surgical procedure is illustrated in Figure 1.

### 2.4 Statistical analysis

Data were analyzed using SPSS software version 23.0. Variables such as operation time, blood loss, drainage tube placement duration, hospital stay, D-J tube placement duration, preoperative and postoperative upper moiety anterior-posterior diameter (APD), upper moiety ureter width, and split renal function of the upper moiety are continuous and were confirmed to be normally distributed through a normal distribution test, the distribution represented as mean ± standard deviation (SD). Comparisons of means between two samples were conducted using an independent samples *t*-test. Age, also a continuous variable, was found to be not normally distributed as determined by the test, and is represented as *M*(*Q*1, *Q*3). A *P*-value <0.05 was considered to indicate statistical significance.

## 3 Results

This study ultimately included 14 children with duplex kidneys (Table 1), consisting of 5 males and 9 females, with a median age of 24.5 months (17.5, 50.0), 10 duplex kidney cases on the left side of the body and 4 on the right. There were 2 cases of febrile urinary tract infections (FUTI), 6 cases of urinary dribbling, and 6 cases were diagnosed with hydronephrosis of the renal pelvis and ureter in the upper moiety during pregnancy ultrasound (US) examination, with progressive worsening of hydronephrosis in both the renal pelvis and ureter observed during follow-up. Preoperative evaluations included urinary system US, MRU, DRS, and voiding cystourethrogram (VCUG). Decisions regarding the preservation of the duplicated upper moiety were based on its morphology as depicted by MRU and the split renal function as indicated by DRS; all 14 upper moiety were distinctly visible on DRS. Among the patients, 7 had ectopic ureteral openings, 4 presented with upper moiety VUR without associated ectopic ureteral openings or ureteral cysts, and the remaining 3 patients had isolated distal ureteral stenosis.

**Table 1:** Clinical data of 14 children.

NO	Gender	Age (months)	Laterality	Preoperative Symptoms	Operative Time (min)	Intraoperative bleeding volume (ml)	Drainage tube retention time (days)	Hospitalization days (days)	D-J tube retention time (days)	Preoperative anterior posterior diameter of upper moiety APD (mm)	Postoperative upper moiety APD (mm)	Preoperative upper moiety ureteral width (mm)	Postoperative upper moiety ureteral width (mm)	Preoperative upper moiety function (%)	Postoperative upper moiety function (%)
1	M	38	L	UI	135	8	3	4	48	25.9	9.1	17.7	4.6	17.7	20.3
2	M	53	L	UI	142	10	3	5	39	13	8.3	9.5	5.2	9.5	12.6
3	F	104	L	FUTI	108	6	4	5	46	28.5	4.5	11.4	4.4	11.4	14.7
4	M	10	R	Pregnancy US	112	6	3	5	43	24	10	12.5	4.2	12.5	17.4
5	F	63	L	FUTI	102	8	3	5	52	26.5	5.4	8.9	5.2	8.9	13.8
6	M	16	L	Pregnancy US	118	5	3	4	45	12.5	6.2	11.7	3.8	11.7	20.2
7	M	27	L	UI	154	5	4	5	36	31	8.4	14.4	4	14.4	16.8
8	M	39	L	UI	122	7	2	4	50	21	7.5	11.5	6.5	11.5	18.2
9	F	49	R	Pregnancy US	114	5	3	4	52	23.5	9.4	9.6	5.1	9.6	14.9
10	M	15	R	Pregnancy US	159	8	4	5	32	16.5	6.3	12.6	3.9	12.6	15.7
11	F	19	L	Pregnancy US	164	10	2	4	48	36	4.8	8.7	5.2	8.7	16.8
12	F	22	R	Pregnancy US	114	15	4	5	39	22.4	7.2	14.7	3.8	14.7	18.5
13	M	18	L	UI	102	5	3	6	44	14.5	3.6	18.3	4.9	18.3	19.5
14	M	21	L	UI	156	8	3	6	36	38.4	3.2	10.4	3.8	10.4	11.6

UI, urinary incontinence; FUTI, fever urinary tract infection; Pregnancy US, pregnancy ultrasound.

**Figure 2:**
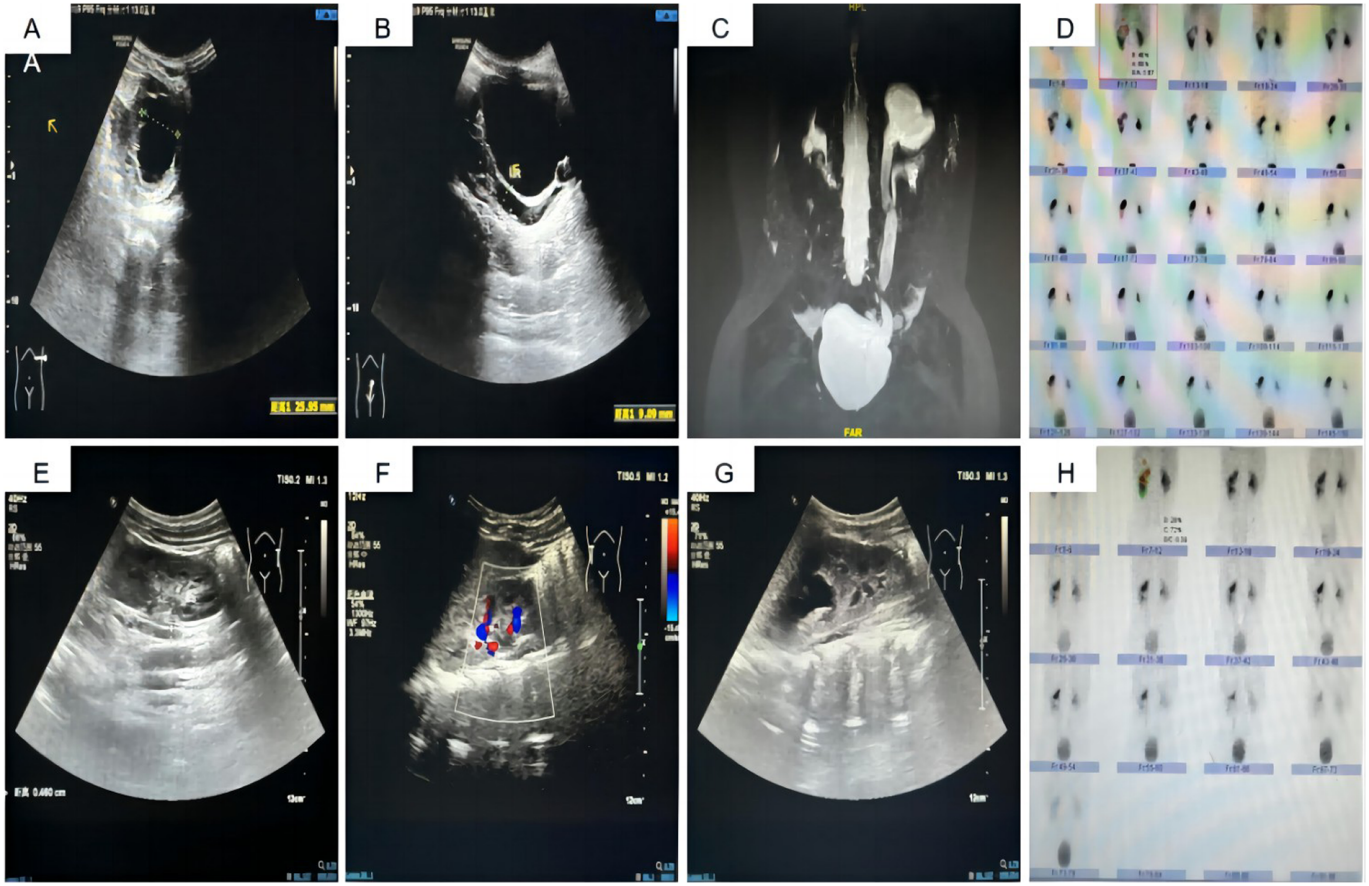
Preoperative and Postoperative Imaging Data. **(A)** Preoperative US displaying the APD of the left upper kidney at 25.9 mm. **(B)** Preoperative US showing the width of the left upper kidney ureter at 9.1 mm. **(C)** Preoperative MRU depicting deformity of the left renal pelvis and ureter, along with hydronephrosis in the left upper kidney and ureter. **(D)** Preoperative DR indicating the split renal function of the left upper kidney at 17.7%. **(E, F)** Postoperative US revealing the left upper kidney APD reduced to 12.8 mm. **(G)** Postoperative US showing the left upper kidney ureter width reduced to 4.6 mm. **(H)** Postoperative DR indicating an increase in split renal function of the left upper kidney to 20.3%.

All 14 surgeries were completed successfully without the need to transition to open surgery. The operation time averaged 128.71 ± 22.35 min, intraoperative blood loss was 7.57 ± 2.77 ml, drainage tube placement duration averaged 3.14 ± 0.66 days, hospital stays were 4.79 ± 0.70 days, and stent placement lasted 43.58 ± 6.33 days. Six months post-surgery, follow-up US and DRS demonstrated significant improvements in preoperative and postoperative upper moiety APD (23.84 ± 8.05 mm vs. 6.71 ± 2.20 mm, *P* < 0.001), diameter at the widest part of the upper moiety ureter (15.58 ± 6.07 mm vs. 4.61 ± 0.78 mm, *P* < 0.001), and split renal function of the upper moiety (12.28 ± 3.04% vs. 16.50 ± 2.75%, *P* < 0.001). These results underscore substantial improvements in kidney hydronephrosis and ureter width post-surgery, alongside an increase in split renal function. None of the 14 children experienced urinary leakage postoperatively; the drainage tube and urinary catheter were removed 3–5 days after surgery, and the D-J stents were extracted under cystoscopy between 32 and 52 days post-surgery. Following discharge, the children received oral Cefaclor (7 mg/kg thrice daily) for one week, followed by a prophylactic dose (7 mg/kg once nightly) until the removal of the D-J stent. During the period with the D-J stent, one child developed a urinary tract infection, which improved with anti-infection treatment. Additionally, one child experienced recurrent gross hematuria, and another exhibited frequent and significant urinary irritation symptoms, with an abdominal plain film revealing that the D-J stent had descended completely into the bladder; symptoms resolved following stent removal. None of the 14 children suffered from anastomotic obstruction, vesicoureteral reflux, stump syndrome, or abdominal pain. Preoperative and postoperative data are illustrated in Table 2 and Figure 2.

**Table 2:** Preoperative and postoperative comparison.

Variables	Preoperative	Postoperative	*t* Value	*P* Value
Upper moiety APD (mm)	23.84 ± 8.05	6.71 ± 2.20	=7.26	<0.001*
Upper moiety ureteral width (mm)	15.58 ± 6.07	4.61 ± 0.78	=7.24	<0.001*
Upper moiety function (%)	12.28 ± 3.04	16.50 ± 2.75	=−6.84	<0.001*

*P < 0.05.

## 4 Discussion

Most patients with duplex kidneys are asymptomatic and may be incidentally discovered during prenatal examinations or postnatal physical assessments, though some exhibit symptoms such as recurrent urinary tract infections, urinary dribbling between normal urination periods, and abdominal pain, among others. According to the Weigert-Meyer law, the upper moiety ureter of a complete duplex kidney has a lower ectopic opening (outside the bladder), making ectopic ureteral openings more likely, with a higher incidence in females than males. When the ectopic opening occurs in girls, it is located distal to the sphincter, often causing intermittent dribbling of urine between normal voiding episodes (7, 8). In this study, seven cases involved ectopic ureteral openings, six of which presented as intermittent dribbling of urine during normal voiding. For infants and children, serious complications predominantly consist of recurrent urinary tract infections, which can significantly impair kidney function and typically necessitate surgical intervention (9).

The transurethral incision (TUI) of ureteroceles in duplex kidneys has long been a topic of debate due to the difficulty in achieving a balance between effective decompression and the risk of postoperative cyst recurrence or newly developed VUR (10). Sander et al. (11) reported that new-onset VUR into the ipsilateral upper moiety occurred in 43.7% of intravesical and 68.7% of extravesical duplex kidney ureterocele cases following TUI, accounting for 56.2% of all duplex kidney ureterocele patients. Additionally, 67.7% of intravesical duplex kidney ureterocele patients and 80.8% of extravesical duplex kidney ureterocele patients required secondary surgical intervention. In our retrospective analysis, we did not observe any cases requiring IUU surgery due to persistent obstruction or newly developed reflux following ureterocele incision. Based on these findings, we suggest that TUI is a minimally invasive and effective first-line treatment for duplex kidneys with obstructive ureteroceles. In cases where obstruction persists or new-onset VUR arises post-TUI, IUU can serve as a viable secondary option.

Chacko et al. (12) emphasized the advantages of IUU in managing duplex kidneys, including those with associated ureteroceles, highlighting its utility in a wide range of duplex ureter dilatations. He further challenged the traditional view that obstructed nonfunctional upper poles necessitate heminephrectomy, arguing that the majority of patients do not exhibit dysplastic kidneys. However, some researchers have raised concerns that retaining nonfunctional segments of duplex kidneys may increase the risk of hypertension, proteinuria, and malignancy over time. Historically, heminephrectomy was the predominant surgical approach for treating duplex kidneys (13). However, the extensive dissection required during kidney removal can lead to functional impairment or even loss of function in up to 5% of patients' lower moiety (14). For children with duplex kidneys considered valuable for preservation, we advocate for nephron-sparing treatments. The primary surgical methods to maintain kidney function include IUU and Ureteral Reimplantation (UR). In this report, all 14 cases were treated with the IUU procedure. Chu et al. (15) analyzed IUU and UR surgeries, observing that both techniques offer benefits and limitations; however, the IUU procedure is associated with less trauma and facilitates a swifter recovery after surgery. Based on our previous surgical experience with IUU and UR, we contend that IUU surgery minimizes the risk of vascular and nerve injuries during the bladder incision needed for reimplantation and also reduces the likelihood of complications such as anastomotic obstruction or vesicoureteral reflux, which can arise from an anti-reflux response during ureteral implantation.

IUU, as one of the techniques for preserving duplex kidneys, is being increasingly adopted by clinicians and has been validated as a safe and effective surgical option (16, 17). Kawal et al. (18) conducted IUU surgery on 53 children with duplex kidneys, categorizing them into groups with upper moiety split renal function greater than 10% (32 cases) and less than 10% (21 cases). The outcomes demonstrated no significant differences in the alleviation of urinary tract dilation and postoperative complications between the groups, affirming that IUU surgery remains a reliable and effective technique for children with upper moiety split renal function below 10%. In our study, 4 of the 14 cases had upper moiety split renal functions below 10%, specifically 9.5%, 8.9%, 9.6%, and 8.7%. Six months following surgery, their split renal functions improved to 12.6%, 13.8%, 14.9%, and 16.8% respectively, and US examinations indicated a marked decrease in renal and ureteral hydronephrosis compared to preoperative levels. For the other 10 cases with preoperative upper moiety split renal function above 10%, postoperative split renal functions also exhibited an upward trend.

The surgical management of IUU has progressed from open surgery to laparoscopic surgery and, more recently, to robot-assisted laparoscopic (RAL) surgery. Regardless of the surgical technique employed, success rates for IUU consistently range from 95% to 100% (4, 5, 19, 20). Tao et al. (4) reported 30 cases of IUU, including 20 laparoscopic surgeries with an average operative time of 178.8 ± 60.71 min, demonstrating no significant difference compared to 10 open surgeries. However, laparoscopic surgery demonstrated advantages in terms of reduced intraoperative blood loss (4.3 ± 0.92 ml), earlier removal of drainage tubes, and shorter postoperative hospital stays compared to open surgery. In contrast, our data revealed that the mean operative time for RAL-IUU was significantly shorter at 128.71 ± 22.35 min, albeit with slightly greater intraoperative blood loss (7.57 ± 2.77 ml). The reduced operative time of RAL-IUU is likely attributable to the advanced robotic surgical platform, which provides enhanced dexterity, a three-dimensional surgical field, and superior capabilities for intracorporeal suturing and knot-tying.

Casale et al. (19) reported successful outcomes in 15 pediatric cases of RAL-IUU, with a mean operative time of 1.2 h, demonstrating the feasibility and safety of this technique in the pediatric population. However, achieving shorter operative times is often contingent on the surgeon's proficiency with robotic systems. Similarly, Lee et al. (5) compared 25 cases of RAL-IUU with 19 cases of open surgery and found no significant differences in operative time (158.0 ± 48.5 min for RAL-IUU) or intraoperative blood loss (6.4 ± 5.6 ml). Both groups exhibited comparable complication rates. The primary distinction was in the anastomosis location, with open surgery favoring distal anastomoses near the bladder and RAL-IUU primarily utilizing proximal anastomoses. IUU may be conducted with an end-to-side anastomosis at the pelvis level or an anastomosis between the upper segment of the ureter at the renal pelvis level and the lower moiety pelvis (20). Typically, the selection of the anastomosis site relies heavily on the surgeon's experience and preference (21). For this procedure, we opted for the IUU approach at the level of the iliac vessels because an end-to-side anastomosis at this site circumvents excessive colon immobilization, spares the gonadal vessels, and obviates the need for significant removal of both the upper portion and much of the remaining distal ureter. This strategy reduces the risk of compromising the blood supply to the lower moiety ureter when the ureters are joined, potentially preventing complications like anastomotic obstruction. Moreover, since it avoids extensive upper moiety ureter removal, it maintains the option for future IUU or UR surgeries. Some studies (22) discuss the possible postoperative “yo-yo” reflux, which could lead to recurrent infections in the recipient ureter and kidney, although this hypothesis has not been substantiated in serial reports. Wong et al. (23, 24) explored laparoscopic pelvic IUU treatments for duplex kidneys, observing no instances of “yo-yo” reflux. They suggest that conducting IUU at the pelvic brim level, where the distal part of the recipient ureter is shorter before joining the bladder, helps avert such complications.

Herz et al. (20) reported on a cohort of pediatric patients with duplicated renal anomalies undergoing robotic surgery, comparing outcomes with open and laparoscopic approaches. Among 47 children, 45 underwent RAL procedures, including 19 cases of RAL heminephrectomy (RAL-HN), 14 cases of RAL-IUU, and 12 cases of RAL common-sheath ureteral reimplants (RAL-csUN) for vesicoureteral reflux and urinary tract infections. The success rates for RAL-HN, RAL-IUU, and RAL-csUN were 94%, 100%, and 85%, respectively, with complication rates of 6%, 7%, and 15%, and mean operative times of 209, 212, and 222 min, respectively. They concluded that robot-assisted surgery for upper urinary tract reconstruction in children with duplicated kidneys is safe, with overall success rates and complication profiles comparable to those of open and laparoscopic approaches, while operative times were similar to laparoscopic procedures.

In summary, RAL-IUU represents a safe and effective alternative to both open and laparoscopic IUU, with comparable surgical outcomes. The advantages of RAL-IUU include shorter operative times compared to laparoscopic surgery and superior cosmetic results owing to its minimally invasive nature when compared to open surgery. Furthermore, the robotic system enhances the surgeon's operative experience by providing improved dexterity and precision. In China, the total number of duplicated kidney malformations malformations in children is relatively high due to the large population, but the adoption of robotic surgeries remains limited by their significant costs. As noted by Mao et al. (25), the high expense of robotic surgery poses significant challenges for families in economically underdeveloped regions. Therefore, selecting the appropriate surgical approach requires careful consideration of the patient's individual circumstances and the availability of medical resources.

From our experience with 14 cases of RAL-UU, we have garnered several important insights: (1) the preoperative insertion of a D-J stent via cystoscopy into the normal ureter facilitates the identification and protection of the ureter; (2) when employing electrocautery to access the paracolic gutter, it's crucial to carefully monitor the direction and maintain a safe distance from the colon to minimize any adverse effects; (3) one of the main challenges in performing end-to-side ureteroureterostomy is the creation of the anastomotic site. Longitudinal incisions in the lower pole of the ureter require meticulous precision to avoid compromising the blood supply. During the procedure, it is essential to minimize the use of clamps on the ureter to prevent damage to the posterior wall. The presence of a D-J stent typically facilitates more precise ureteral incisions. For novice surgeons, a transabdominal suspension technique may be employed, where a 7-0 suture is used to suspend and apply tension to the outer wall of the ureter, stabilizing the segment to be incised. This ensures a stable position before making the sidewall incision of the normal ureter (17, 21). We initially applied this technique in the first five cases, and after achieving proficiency, stabilization of the distal ureter no longer required this method; (4) anastomosis is critical for successful surgery. While some research suggests that ureteroureterostomy may not be advisable for severely dilated ureters, definitive evidence is still lacking (26). However, other studies have confirmed that performing IUU after appropriately trimming and reducing the size of the ureteral orifice is safe and effective (3, 18, 27). In our research, three cases had a preoperative ureter diameter greater than 20 mm, all showing significant improvement in renal pelvis and ureteral hydronephrosis post-surgery without any subsequent urinary tract infections. It is crucial to select the appropriate site for anastomosis to avoid ureteral twisting and ensure a smooth, tension-free connection; (5) Robotic surgery facilitates a broader surgical view in the pelvis area, making it advisable to ligate the upper moiety ureter as distally as possible.

## 5 Conclusion

Our initial experience suggests that RAL-IUU is a safe and effective surgical alternative for children with complete duplex kidneys. This study, however, encompasses only a limited number of cases and the follow-up duration has been brief. An expansion in the number of cases and an extension of the follow-up period are essential to derive more reliable and robust conclusions.

## Data Availability

The datasets presented in this study can be found in online repositories. The names of the repository/repositories and accession number(s) can be found in the article/Supplementary Material.
